# Design and Implementation of a Brief, Self-Directed Course on Immunotherapy Best Practices for Neurology Trainees

**DOI:** 10.1177/23821205241271546

**Published:** 2024-08-09

**Authors:** Kyle M. Blackburn, Peter V. Sguigna, Lauren Tardo, Shaida Khan, Steven Vernino, Lauren Phillips

**Affiliations:** 1 Department of Neurology, 12334 University of Texas Southwestern Medical Center, Dallas, TX, USA; 2 171786 Peter O’Donnell Jr. Brain Institute, University of Texas Southwestern Medical Center, Dallas, TX, USA

**Keywords:** medical education, neuroimmunology, disease-modifying therapy

## Abstract

**OBJECTIVES:**

To create and implement a brief, self-directed course on immunotherapy (IT) best practices for trainees on a neuroimmunology elective rotation.

**METHODS:**

A working group of neurology faculty developed a curriculum covering the mechanism of action, indications, and necessary monitoring for different IT used in neurology practice. The content was presented as a web-based course and hosted on local servers. Neurology residents and fellows participating in a neuroimmunology elective were given access to the curriculum over a 2-week period. A multiple-choice assessment and questionnaire assessing learner confidence with IT was administered prior to starting the course, and again upon course completion. Twelve months after implementation, the pretest and posttest were revised following an item analysis.

**RESULTS:**

Twenty-two neurology residents and fellows completed the course since July 2022. The average score on the first version of the pretest and posttest was 78% versus 92% (*P *= .02), and 51% versus 70% (*P =* .02) on the revised version. Trainee self-reported confidence with IT also improved, although only 59.1% of trainees completed the postcourse questionnaire. Respondents provided positive feedback on the format and content of the course and expressed a desire for a reference to the material for future use.

**CONCLUSION:**

In this pilot study, our course improved resident confidence and knowledge of IT best practices. The course was well-received, and our methods can be implemented in a variety of clinical environments to supplement trainee learning.

## Introduction

Immunotherapies (IT) are commonly used in neurology practice in the inpatient and outpatient setting. While neurologists are well versed in the use of glucocorticoids, intravenous immunoglobulins, and plasma exchange, the number of maintenance therapies available for multiple sclerosis (MS), neuromyelitis optica spectrum disorders, and other neuroimmunological disorders have rapidly expanded in the past decade.^1–3^ The use of IT increases the risk of opportunistic infections, malignancy, or secondary autoimmune disorders, and close monitoring tailored to the specific therapy is required to prevent complications.

Traditionally, neurology trainees have acquired knowledge of IT through direct clinical contact, with limited time dedicated to education on the mechanism of action, indications, and necessary safety monitoring. Strategies such as oral lectures are a common teaching method in resident education, but are typically asynchronous with a trainee's clinical exposure, and often fail to emphasize rational prescribing principles essential to clinical practice.^4,5^ Self-directed curricula, often administered via web-based modules, have emerged as a promising method to deliver such interventions as they can be incorporated into clinical rotations as a blended learning strategy.^6–9^ In neurology, self-directed curricula have been used to educate learners on the principles of electrophysiology and the neurological exam with favorable results.^8,10–15^ Here, we describe the development and integration of a self-directed, web-based course (Safe Practice with Immunotherapies in Neurology [SPIN]) for neurology residents and fellows into an outpatient elective at the University of Texas Southwestern Medical Center (UTSW).

## Objectives

The objectives of the SPIN course were to (a) provide education on best practices for the safe use of IT used in neurology; (b) offer the course in a self-paced manner that supplements learning through direct clinical care in the neuroimmunology clinic; and (c) improve the learners’ overall confidence in managing IT in clinical practice.

## Methods

### Course development

A group of 6 neurologists specializing in neuroimmunological disorders of the central or peripheral nervous system participated in developing the SPIN curriculum. A comprehensive list of IT used in neurology practice was compiled. For each therapy, the mechanism of action, route of administration, side effects, and appropriate clinical and laboratory monitoring were collected from a review of United States package inserts and pertinent literature. Additional topics applicable to IT safety, such as progressive multifocal leukoencephalopathy and rebound disease activity associated with certain MS therapies, were included in the curriculum. The final SPIN curriculum was divided into 3 sections, titled “Multiple Sclerosis Disease Modifying Therapies,” “Acute Therapies,” and “Other Commonly Used and Emerging Immunotherapies.” The content and presentation of each course were reviewed by all working group members and revised until a consensus was reached.

One objective of this effort was to offer learners a flexible experience that would accompany learning through clinical care, drawing from blended learning models.^16^ Accordingly, a web-based course was developed using RISE 360 (Articulate, New York, NY), an eLearning platform that allows learners to access content at their own pace, on their preferred device. RISE 360 courses require little experience with web design, and content is formatted for both personal computers and mobile devices. Information was presented in a text format and focused on the mechanism of action, required monitoring, side effects, and safety considerations for each therapy. Where appropriate, elements such as virtual flashcards were used throughout the course to make the course more interactive (see Supplemental File for a copy of the full course). The curriculum, tests, and questionnaires were hosted on a Microsoft SharePoint site available on local servers.

### Recruitment and study population

Neurology residents participating in a 2-week neuroimmunology elective at UTSW were invited to participate in the course starting in academic year 2022 (AY 2022; July 2021–June 2022). Residents may participate in the elective at any point during their residency, including their intern year. Traditionally, our center provides IT education through direct clinical contact during their clinical rotations, in addition to a 1-h lecture on disease-modifying therapy for MS delivered during resident didactics, approximately once every other year. Neuroimmunology fellows were invited to complete the course during their first month of fellowship as it offers foundational knowledge for their training. Completion of the course was optional, and residents could complete the course at any point during the elective.

### Outcome measures

To assess knowledge acquisition, pretest and posttest were developed, each including 10 unique multiple-choice questions (see Supplemental File). An item bank for each test was developed to cover 4 knowledge domains: baseline/screening requirements, follow-up monitoring, side effects/adverse events, and pregnancy/family planning. Each pretest and posttest was composed of a balanced number of questions from each domain: baseline/screening requirements (5 items), follow-up monitoring (2), side effects/adverse events (2), and pregnancy (1).

Precourse and postcourse questionnaires were administered following each test. Demographic information, including specialty (adult or child neurology), and postgraduate training year (PGY), was collected in the precourse questionnaire. Both precourse and postcourse questionnaires included questions on trainees’ perceived confidence with IT rated on an ordinal 5-point Likert scale (options ranging from 1 “Very uncomfortable” to 5 “Very comfortable”). To gather feedback on the course, the postcourse questionnaire included statements on the content and presentation of the lessons, and trainees rated their agreement on a Likert scale (ranging from 1 “strongly disagree” to 5 “strongly agree”). A free text question soliciting any additional comments or feedback was included to gather additional trainee perspectives.

### Statistical analysis

An item analysis was performed on questions in the pretest and posttest at the end of AY 2022 by calculating the percent-correct value and discrimination index (biserial correlation coefficient) for each question. Results of the pretest and posttest were compared using a paired *t*-test. The weighted average for precourse and postcourse confidence levels was calculated, and matching precourse and postcourse questionnaires were compared using a paired *t*-test. Statistical analysis was performed in Microsoft Excel using the Data Analytics package.

### Ethical approval

The local Institutional Review Board reviewed this protocol and determined this study to be exempt from full board review.

### Participant consents

The requirements for informed consent were waived by the Institutional Review Board, and participation in the course was voluntary.

### Data availability

Anonymized data not published in this article will be made available at the request of any qualified investigator.

## Results

Since the course was launched in July 2022, 27 trainees were invited to complete the course, and 22 accessed the content (81.4% participation). Residents in their PGY-2 and PGY-4 levels of training comprised most participants in the course (68.2%) (Table 1). Eighteen respondents (81.8%) reported ordering IT (other than glucocorticoids, intravenous immunoglobulin, or plasma exchange) during the previous 6 months.

**Table 1.** Demographic information.

LEVEL OF TRAINING	PERCENTAGE OF RESPONDENTS (*n* = 22)
PGY-1 (resident)	13.6
PGY-2 (resident)	36.3
PGY-3 (resident)	13.6
PGY-4 (resident)	31.8
PGY-5 (fellow)	4.5
Estimated use of IT over a 6-month period (%)	
0 instances	18.2
1–5 instances	63.6
6–10 instances	18.2

Abbreviations: PGY, postgraduate year; IT, Immunotherapy.

### Knowledge pretest and posttest scores

Nineteen trainees completed the pretest and posttest since the course was implemented. In AY 2022, 10 trainees completed the tests, averaging 78% on the pretest, and 92% on the posttest (*P = *.02). Following item analysis prior to AY 2023, 6 questions on both pretest and posttest were revised or replaced (Supplemental Table 1). Since AY 2023, 9 trainees have completed the new versions of the tests. Test scores improved from 51% on the pretest versus 70% on the posttest (*P = *.02). Repeat item analysis on the new tests demonstrates improved discrimination indices (Supplemental Table 2).

### Precourse and postcourse confidence

**Figure 1.**
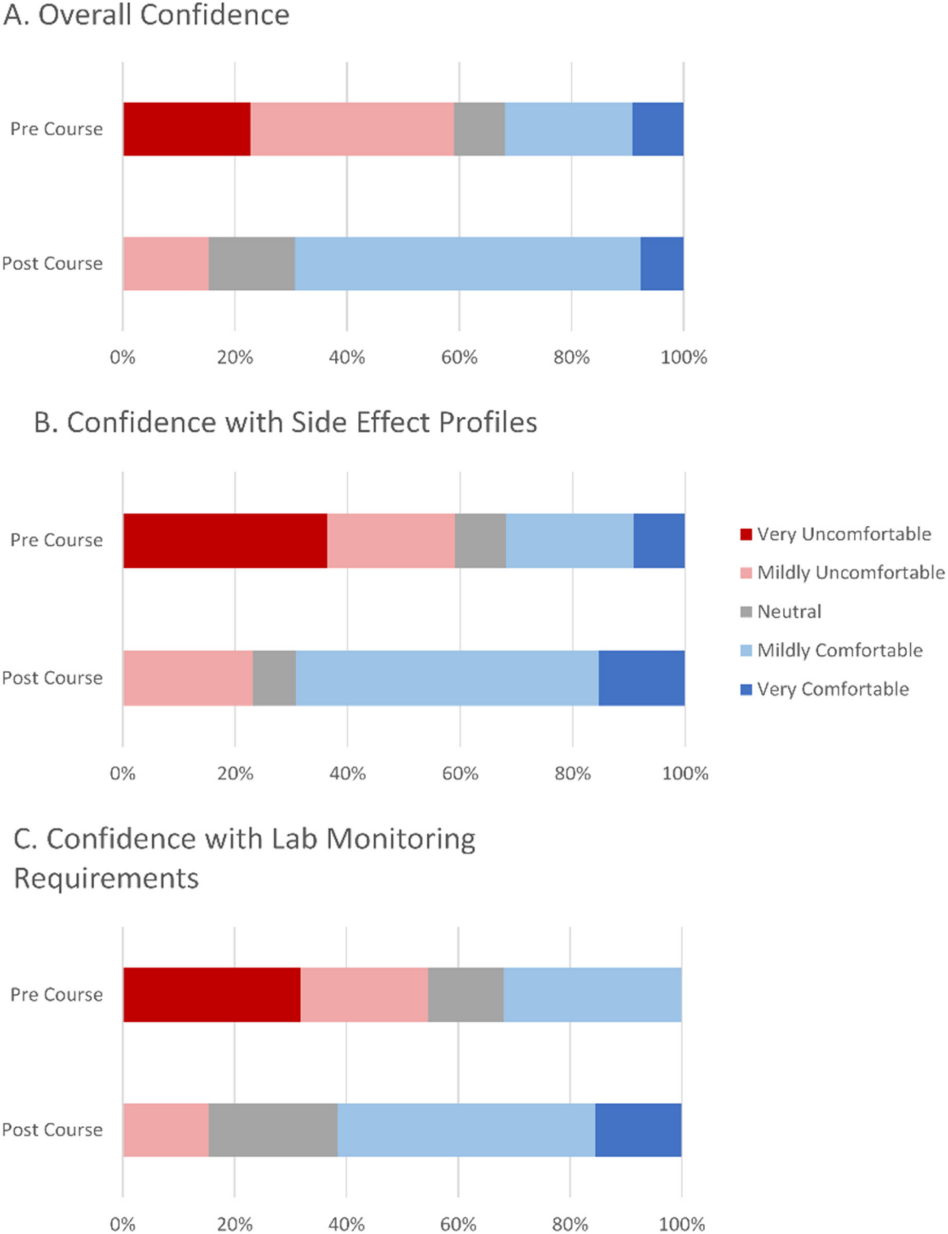
Trainee confidence with immunotherapy prescribing improved following course completion: (A) overall confidence, (B) confidence with side effect profiles, and (C) confidence with lab monitoring requirements.

In the precourse questionnaire, 68.2% of participants rated their overall comfort with managing IT as very uncomfortable, mildly uncomfortable, or neutral (Figure 1). The same proportion reported feeling uncomfortable with their knowledge of side effects and monitoring requirements of IT. Thirteen trainees (59.1% of total participants) completed the postcourse questionnaire. The average overall confidence with IT improved from 2.59 to 3.61 following the course. Among those with complete data, confidence increased from 2.31 to 3.62 upon completing the course (*P = *.0001).

### Trainee feedback

**Figure 2.**
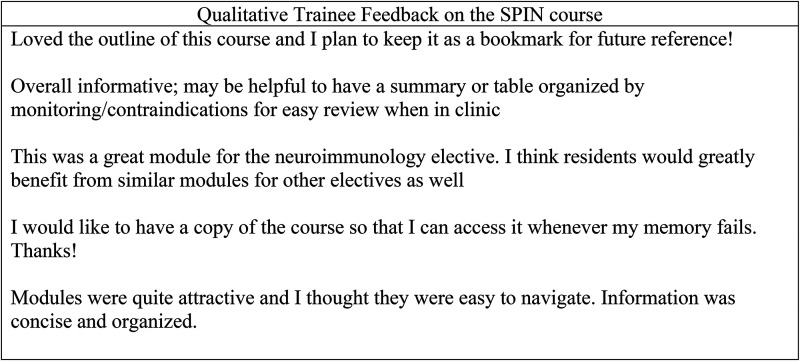
Examples of qualitative feedback from trainees on the postcourse questionnaire.

In the postcourse questionnaire, trainees rated their agreement with statements about the educational content and delivery of the course. Learners all agreed that the course improved their knowledge about IT and that the course was engaging and easy to navigate. Trainees expressed strong interest in the web-based format for future courses. Qualitative feedback on the course was favorable, with positive comments about the design and outline of the material (Figure 2). Trainees frequently requested a reference of the material for future access to review specific material during clinical care.

## Discussion

As therapeutic options for neuroimmunological disorders grow, there is a need for dedicated curricula to familiarize neurologists with important adverse events and monitoring requirements associated with IT. To fill this gap, we developed a web-based course on IT best practices that was administered during our neuroimmunology elective. The SPIN course was easily developed and implemented and improved confidence and immediate knowledge acquisition among trainees.

The rising generation of medical learners gained access to the internet and mobile devices at a young age and interacted with digital content daily.^17^ Accordingly, current learners prefer educational content that is available electronically and allows for self-paced learning.^18,19^ To adapt to these preferences, national organizations have increased their eLearning offerings, and medical educators have developed online curricula geared toward neurology trainees.^8,14,20–22^ Although preliminary, our intervention highlights some potential benefits of using online courses in a blended learning model accompanying a clinical elective. Specifically, our course provided targeted education on IT while trainees were seeing the treatments used in clinical encounters, which likely has synergistic benefits for learning. Our methods can be easily applied to create blended learning curricula and may be particularly helpful for residents during subspecialty rotations.

Although promising, blended and self-directed learning strategies have significant limitations. Self-directed learning strategies require significant motivation for learners, as they must dedicate time and energy to reviewing the material outside the clinical environment. Thus, this approach is not ideal for environments with heavy cognitive loads, such as those with high clinical volumes and long hours. There are also technological limitations to this approach, as blended learning requires learners to have access to high-quality devices and the Internet. Likewise, educators must have access to appropriate software and be motivated to overcome the learning curve associated with web design, especially if interactive elements such as video are planned.

While learners responded positively to the format of our course, it is currently primarily text-based, which limits learner engagement. In the future, we plan to address this by adding knowledge checks and clinical scenarios to the course to promote critical thinking throughout and emphasize the applicability of the information. The addition of short videos would also make our course more engaging and may improve knowledge retention for certain topics. We also recognize the importance of repetition of the information for knowledge retention, and our trainees identified the need for a reference that can be accessed in clinical settings. The best approach to implement this is being explored.

Our pilot study has several limitations. First and foremost, our sample size is small compared to other studies investigating curricula in medical education.^9,23,24^ This is largely attributed to the size of our available population, as our total complement at the time of curriculum launch was 36 residents. In the future, offering our curriculum at multiple programs would increase our sample size and test the efficacy of our intervention in different learning environments. Including neuroimmunology fellows, who have a deeper exposure to neuroimmunology and higher incentives to learn the material, could bias our results. We feel the information is particularly important for fellows and we encouraged them to complete the course in the first month of training, when their baseline exposure to neuroimmunology is comparable to a senior resident. Although this was discouraged in the course instructions, trainees may have accessed the course during the posttest, which may confound our posttest results. Our learning management system also did not allow us to determine how much time learners spent in the course, or if they visited the course repeatedly throughout the elective; such data will be helpful in determining how learners are interacting with the course. As our posttest assessment was often completed immediately upon course completion, we were unable to assess delayed retention of the knowledge of our course. In addition, our tests and questionnaires were not validated or pilot-tested prior to implementation given the small number of participants available in the study population. Just over half of our trainees completed the postcourse questionnaire, introducing the potential for bias due to attrition error as subjects that did not complete the questionnaires may have negative impressions of the course or lower confidence levels.

## Conclusion

In conclusion, our pilot study suggests our web-based course was well-received and feasible to implement during a clinical elective. Larger studies are needed to assess its applicability across different learning environments.

## Supplemental Material

sj-docx-1-mde-10.1177_23821205241271546 - Supplemental material for Design and Implementation of a Brief, Self-Directed Course on Immunotherapy Best Practices for Neurology Trainees Supplemental material, sj-docx-1-mde-10.1177_23821205241271546 for Design and Implementation of a Brief, Self-Directed Course on Immunotherapy Best Practices for Neurology Trainees by Kyle M. Blackburn, Peter V. Sguigna, Lauren Tardo, Shaida Khan, Steven Vernino and Lauren Phillips in Journal of Medical Education and Curricular Development

sj-docx-2-mde-10.1177_23821205241271546 - Supplemental material for Design and Implementation of a Brief, Self-Directed Course on Immunotherapy Best Practices for Neurology Trainees Supplemental material, sj-docx-2-mde-10.1177_23821205241271546 for Design and Implementation of a Brief, Self-Directed Course on Immunotherapy Best Practices for Neurology Trainees by Kyle M. Blackburn, Peter V. Sguigna, Lauren Tardo, Shaida Khan, Steven Vernino and Lauren Phillips in Journal of Medical Education and Curricular Development

sj-docx-3-mde-10.1177_23821205241271546 - Supplemental material for Design and Implementation of a Brief, Self-Directed Course on Immunotherapy Best Practices for Neurology Trainees Supplemental material, sj-docx-3-mde-10.1177_23821205241271546 for Design and Implementation of a Brief, Self-Directed Course on Immunotherapy Best Practices for Neurology Trainees by Kyle M. Blackburn, Peter V. Sguigna, Lauren Tardo, Shaida Khan, Steven Vernino and Lauren Phillips in Journal of Medical Education and Curricular Development

sj-docx-4-mde-10.1177_23821205241271546 - Supplemental material for Design and Implementation of a Brief, Self-Directed Course on Immunotherapy Best Practices for Neurology Trainees Supplemental material, sj-docx-4-mde-10.1177_23821205241271546 for Design and Implementation of a Brief, Self-Directed Course on Immunotherapy Best Practices for Neurology Trainees by Kyle M. Blackburn, Peter V. Sguigna, Lauren Tardo, Shaida Khan, Steven Vernino and Lauren Phillips in Journal of Medical Education and Curricular Development

sj-docx-5-mde-10.1177_23821205241271546 - Supplemental material for Design and Implementation of a Brief, Self-Directed Course on Immunotherapy Best Practices for Neurology Trainees Supplemental material, sj-docx-5-mde-10.1177_23821205241271546 for Design and Implementation of a Brief, Self-Directed Course on Immunotherapy Best Practices for Neurology Trainees by Kyle M. Blackburn, Peter V. Sguigna, Lauren Tardo, Shaida Khan, Steven Vernino and Lauren Phillips in Journal of Medical Education and Curricular Development
