# Supplementary material for: Design and Implementation of a Brief, Self-Directed Course on Immunotherapy Best Practices for Neurology Trainees
Source: J Med Educ Curric Dev. 2024 Aug 9;11:23821205241271546. doi: 10.1177/23821205241271546 (PMC11311178; doi:10.1177/23821205241271546)
Supplement: sj-docx-1-mde-10.1177_23821205241271546 - Supplemental material for Design and Implementation of a Brief, Self-Directed Course on Immunotherapy Best Practices for Neurology Trainees [file sj-docx-1-mde-10.1177_23821205241271546.docx]

To Access the course, please enter the following URL into a web browser:

https://rise.articulate.com/author/IRFZAjyf8qexYqjcis4WbI8bHriLAWBD#/author/course
