# Supplementary material for: Design and Implementation of a Brief, Self-Directed Course on Immunotherapy Best Practices for Neurology Trainees
Source: J Med Educ Curric Dev. 2024 Aug 9;11:23821205241271546. doi: 10.1177/23821205241271546 (PMC11311178; doi:10.1177/23821205241271546)
Supplement: sj-docx-2-mde-10.1177_23821205241271546 - Supplemental material for Design and Implementation of a Brief, Self-Directed Course on Immunotherapy Best Practices for Neurology Trainees [file sj-docx-2-mde-10.1177_23821205241271546.docx]

| Pretest | | | | | | | | | | |
| --- | --- | --- | --- | --- | --- | --- | --- | --- | --- | --- |
| Question | 1 | 2 | 3 | 4 | 5 | 6 | 7 | 8 | 9 | 10 |
| Difficulty  (% correct value) | 70 | 100 | 100 | 100 | 50 | 90 | 80 | 60 | 90 | 40 |
| Discrimination index | 0.79 | 0 | 0 | 0 | 0.34 | 0.71 | 0.79 | 0.71 | 0.15 | 0.67 |
| Changes | None | Replace | Revise | Revise | None | Revise | Revise | None | Replace | None |
| Posttest | | | | | | | | | | |
| Question | 1 | 2 | 3 | 4 | 5 | 6 | 7 | 8 | 9 | 10 |
| Difficulty  (% correct) | 90 | 100 | 100 | 100 | 50 | 100 | 100 | 100 | 90 | 90 |
| Discrimination index | 0.09 | 0 | 0 | 0 | 0.80 | 0 | 0 | 0 | 0.53 | 0.53 |
| Changes | None | Replace | Revise | Replace | None | Replace | Revise | Revise | None | None |

Supplemental Table 1. Item analysis of pretest and posttest with changes for tests administered in AY 2022. Percentage-correct values of 80 or greater and discrimination index <0.4 were considered for revision by the working group.

| Pretest | | | | | | | | | | |
| --- | --- | --- | --- | --- | --- | --- | --- | --- | --- | --- |
| Question | 1 | 2 | 3 | 4 | 5 | 6 | 7 | 8 | 9 | 10 |
| Difficulty  (% correct value) | 50 | 75 | 50 | 50 | 50 | 25 | 58 | 17 | 58 | 41 |
| Discrimination index | 0.48 | 0.71 | 0.37 | 0.60 | 0.71 | 0.61 | 0.80 | 0.29 | 0.69 | 0.70 |
| Posttest | | | | | | | | | | |
| Question | 1 | 2 | 3 | 4 | 5 | 6 | 7 | 8 | 9 | 10 |
| Difficulty  (% correct) | 89 | 89 | 44 | 67 | 56 | 22 | 78 | 100 | 67 | 89 |
| Discrimination index | 0.53 | 0.53 | 0.34 | 0.94 | 0.45 | 0.27 | 0.8 | 0 | 0.47 | 0.53 |

Supplemental Table 2. Item analysis of pretest and posttest with changes for second version of pre- and post-tests.
