# Supplementary material for: Design and Implementation of a Brief, Self-Directed Course on Immunotherapy Best Practices for Neurology Trainees
Source: J Med Educ Curric Dev. 2024 Aug 9;11:23821205241271546. doi: 10.1177/23821205241271546 (PMC11311178; doi:10.1177/23821205241271546)
Supplement: sj-docx-3-mde-10.1177_23821205241271546 - Supplemental material for Design and Implementation of a Brief, Self-Directed Course on Immunotherapy Best Practices for Neurology Trainees [file sj-docx-3-mde-10.1177_23821205241271546.docx]

1. While no consensus has been reached, what is the single therapy for multiple sclerosis that many providers consider to be safe for women to continue during pregnancy?

A. Dimethyl Fumarate

B. Interferon

C. Natalizumab

D. Glatiramer Acetate*

E. Teriflunomide

1. Which of the following baseline tests should be obtained prior to initiating mycophenolate mofetil in a woman?
2. Complete Blood count with differential
3. Liver Function Tests

C. Pregnancy test

D. Varicella Zoster Virus Antibody

E. A, B and C*

F. All of the Above

1. A 43 year-old female with relapsing remitting multiple sclerosis presents to the hospital with a three-day history of headache, lethargy, fever, and worsening confusion. She has been on fingolimod for two years without evidence of relapse.  On physical exam she demonstrates nuchal rigidity. Further CSF results are pending. Which infectious agent would be highest on your differential diagnosis?

A. Tuberculosis

B. Cryptococcus*

C. Meningococcus (Neisseria Meningitidis)

D. Listeria Montocytogenes

4. Which of the following therapies does not require routine blood monitoring?

A. Dimethyl Fumarate

B. Interferons

C. Glatiramer Acetate*

D. Natalizumab

5. Which of the following is a side effect of interferons?

A. Depression*

B. Macular Edema

C. Immune mediated thrombocytopenia

D. Pulmonary Fibrosis

6. Which of the following increases the risk for developing progressive multifocal leukoencephalopathy while taking natalizumab?

1. History of prior immunosuppression
2. Positive JC Virus Antibody
3. Receiving natalizumab infusions for 1 year.
4. A and B *
5. All of the above

7. A patient with AQP4+ NMO presents to the clinic and during the visit you both agree to pursue treatment with eculizumab. Which of the following procedures should occur prior to initiation of therapy?

A) Pneumococcal vaccination

B) Meningococcal vaccination*

C) Optical Coherence Tomography

D) Electrocardiogram

8. Which of the following drugs is associated with a risk of developing demyelinating disease in the central or peripheral nervous system?

1. Methotrexate
2. Tacrolimus
3. Cyclophosphamide
4. Infliximab*
5. All of the Above

9. Which of the following therapies does NOT carry an increased risk for skin cancers?

1. Azathioprine
2. Fingolimod
3. Ofatumumab*
4. Tacrolimus
5. Mycophenolate Mofetil

10. A 35 year-old man with multiple sclerosis presents to clinic after completing his second cycle of cladribine. He tolerated the medication well, and plans to complete his required blood work after clinic. You plan to initiate herpes prophylaxis if his absolute lymphocyte count is:

1. 100
2. 300
3. 500
4. 700
5. A or B
