# Supplementary material for: Design and Implementation of a Brief, Self-Directed Course on Immunotherapy Best Practices for Neurology Trainees
Source: J Med Educ Curric Dev. 2024 Aug 9;11:23821205241271546. doi: 10.1177/23821205241271546 (PMC11311178; doi:10.1177/23821205241271546)
Supplement: sj-docx-4-mde-10.1177_23821205241271546 - Supplemental material for Design and Implementation of a Brief, Self-Directed Course on Immunotherapy Best Practices for Neurology Trainees [file sj-docx-4-mde-10.1177_23821205241271546.docx]

1. Which of the following medications carries a risk of rebound disease activity upon cessation of the drug?
2. Natalizumab
3. Fingolimod
4. Teriflunomide
5. A and B*
6. All of the Above
7. Which of the following is a side effect of glatiramer acetate?
8. Lipodystrophy*
9. Hypothyroidism
10. Lymphopenia
11. Transaminitis
12. Flu-like symptoms

3. Of the following infections, which are known to have a higher risk of reactivation with administration of CD20 therapies (rituximab, ocrelizumab, ofatumumab)?

A. Tuberculosis
B. Hepatitis B
C. Varicella Zoster Virus

D. A and B
E. All of the Above*

1. You have administered cyclophosphamide to a patient for CNS vasculitis, and plan to pursue repeat treatments in the following months. The absolute neutrophil count should be at or above which number prior to the next dose?
2. 500
3. 1000
4. 1500
5. 2000
6. Due to potential increased risk of fetal anomalies, it is recommended that which of the following therapies be discontinued in both women AND men prior to conception?

A. Teriflunomide

B. Cladribine

C. Mycophenolate mofetil

D. A and B

E All of the above

1. Your patient with myasthenia gravis has been well managed on eculizumab for the past 3 years, and will remain on treatment. Which of the following vaccinations need to be administered?
2. Meningococcal A,C,W and Y
3. Meningococcal B
4. Varicella Zoster Virus
5. A and B
6. All of the Above

7. Of the following, which are recommended baseline lab tests prior to starting interferon therapy for multiple sclerosis?

A. Liver function tests

B. Complete Blood Counts

C. Thyroid Stimulating Hormone

D A and B

E. All of the above

1. Which of the following is a side effect of dimethyl fumarate:
2. Macular edema
3. Depression
4. Reactivation of hepatitis
5. Lymphopenia
6. Hypothyroidism.
7. Prior to initiating fingolimod, screening for which of the following infections is recommended?
8. Human immunodeficiency Virus (HIV)
9. Varicella Zoster Virus*
10. Tuberculosis
11. All of the Above
12. Of the following oral therapies, which does NOT carry an increased risk of hepatotoxicity?
13. Tacrolimus*
14. Methotrexate
15. Azathioprine
