# Supplementary material for: Design and Implementation of a Brief, Self-Directed Course on Immunotherapy Best Practices for Neurology Trainees
Source: J Med Educ Curric Dev. 2024 Aug 9;11:23821205241271546. doi: 10.1177/23821205241271546 (PMC11311178; doi:10.1177/23821205241271546)
Supplement: sj-docx-5-mde-10.1177_23821205241271546 - Supplemental material for Design and Implementation of a Brief, Self-Directed Course on Immunotherapy Best Practices for Neurology Trainees [file sj-docx-5-mde-10.1177_23821205241271546.docx]

**Defined Criteria To Report INnovations in Education (DoCTRINE)**

*Citation:* Blanco M, Prunuske J, DiCorcia M, Learman LA, Mutcheson B, Huang GC. The DoCTRINE Guidelines: Defined Criteria To Report INnovations in Education. Academic Medicine. 2022 May 1;97(5):689-695.

| **Introduction** | **Y/N** |
| --- | --- |
| 1. Need for the curriculum | **Y** |
| 1. Review of relevant literature, theories, models, or published curricula | **Y** |
| 1. Unique contribution of the curriculum to the literature | **Y** |
| **Curriculum Development** | **Y/N** |
| 1. Purpose/goals of the curriculum | **Y** |
| 1. Outcome-based learning objectives | **Y** |
| 1. Target population of learners | **Y** |
| **Curriculum implementation** | **Y/N** |
| 1. Instructional setting for curriculum delivery | **Y** |
| 1. Resources for implementing the curriculum | **Y** |
| 1. Description of instructional methods | **Y** |
| 1. Methods to evaluate achievement of outcome-based learning objectives | **Y** |
| 1. Origin of evaluation instrument(s) | **Y** |
| **Results** | **Y/N** |
| 1. Number of learners participating in the curriculum | **Y** |
| 1. Number of participants included in the evaluation | **Y** |
| 1. Evidence of achievement of outcome-based learning objectives | **Y** |
| **Discussion** | **Y/N** |
| 1. Summary of findings | **Y** |
| 1. Interpretation of findings in relation to the existing literature | **Y** |
| 1. Lessons learned from the implementation of the curriculum | **Y** |
| 1. Limitations of the evaluation of the curriculum | **Y** |
| 1. Describes future implications of the curriculum | **Y** |
